# Changes in the ganglion cell complex thickness after anti-VEGF treatment for diabetic macular edema

**DOI:** 10.5935/0004-2749.20200045

**Published:** 2024-02-11

**Authors:** Emine Ciloglu, Fikret Unal, Nese Cetin Dogan

**Affiliations:** 1 Department of Ophthalmology, Adana City Training and Research Hospital, Adana, Turkey

**Keywords:** Diabetes mellitus, Macular edema, Ganglion cell complex, Anti-vascular endothelial growth factor, Neurodegenerative diseases, Tomography, optical coherence, Diabetes mellitus, Edema macular, Complexo de células ganglionares, Anti-fator de crescimento vascular endotelial, Doenças neurodegenerativas, Tomografia de coerência óptica

## Abstract

**Purpose:**

To assess tomographic ganglion cell complex changes in patients with diabetic macular edema treated with intravitreal injections of anti-vascular endothelial growth factor (anti-VEGF).

**Methods:**

We analyzed data from 35 eyes of 35 previously untreated patients in whom diabetic macular edema improved after three loading doses of anti-VEGF injection and who did not receive repeated injections. We recorded spectral domain-optical coherence tomography assessments of ganglion cell complex and central macular thickness at baseline and monthly for three months, and on the sixth and ninth month after treatment. We compared the results with those of the unaffected eyes in the same patients and with those in a control group of patients with diabetic macular edema who were untreated.

**Results:**

The mean age of the patients in the treatment group was 60 ± 4.38 years. The foveal thicknesses measured using optical coherence tomography decreased significantly from baseline to the third month post-injection (p<0.05). The mean ganglion cell complex thickness was 115.08 ± 16.72 µm before the first injection and 101.05 ± 12.67 µm after the third injection (p<0.05). The mean ganglion cell complex was 110.04 ± 15.07 µm on the sixth month (p>0.05) and 113.12 ± 11.15 µm on the ninth month (p>0.05). We found a significant difference between the patients and the control group in terms of mean ganglion cell complex thickness on the secondand third-months post-injection (p<0.05).

**Conclusion:**

Our study showed that the ganglion cell complex thickness in patients with diabetic macular edema decreased after the anti-VEGF injections. We cannot ascertain whether the ganglion cell complex thickness decreases were due to effects of the anti-VEGF agents or to the natural disease course.

## Abstract (pt)

**Objetivo:**

Avaliar as alterações do complexo tomográfico das células ganglionares em pacientes com edema macular diabético tratados com injeções intravítreas do fator de crescimento endotelial anti-vascular (anti-VEGF).

**Métodos:**

Analisamos dados de 35 olhos de 35 pacientes previamente não tratados nos quais o edema macular diabético melhorou após três doses de injeção de anti-VEGF e que não receberam injeções repetidas. Registramos avaliações da tomografia de coerência óptica de domínio espectral do complexo de células ganglionares e da espessura macular central na linha de base e mensalmente por três meses e, também no sexto e nono mês após o tratamento. Comparamos os resultados com os olhos não afetados nos mesmos pacientes e com os de um grupo controle de pacientes com edema macular diabético que não foram tratados.

**Resultados:**

A média da idade dos pacientes no grupo de tratamento foi de 60 ± 4,38 anos. As espessuras foveais medidas pela tomografia de coerência óptica diminuiram significativamente desde o início até o terceiro mês após a injeção (p<0,05). A espessura média do complexo de células ganglionares foi de 115,08 ± 16,72 µm antes da primeira injeção e 101,05 ± 12,67 µm após a terceira injeção (p<0,05). A média do complexo de célula ganglionar foi de 110,04 ± 15,07 µm no sexto mês (p>0,05) e 113,12 ± 11,15 µm no nono mês (p>0,05). Encontramos uma diferença significativa entre os pacientes e o grupo controle quanto à média da espessura do complexo de células ganglionares no segundo e terceiro meses após a injeção (p<0,05).

**Conclusão:**

Nosso estudo mostrou que a espessura do complexo de células ganglionares em pacientes com edema macular diabético diminuiu após as injeções de anti-VEGF. Não podemos determinar se a diminuição da espessura do complexo de células ganglionares ocorreu devido aos efeitos dos agentes anti-VEGF ou ao curso natural da doença.

## INTRODUCTION

Diabetic macular edema (DME) is a common cause of vision loss in patients with diabetes^(1)^. Recently, many antiangiogenic drugs have been used to treat DME^(2)^. Usually, a sequence of three intravitreal injections of anti-vascular endothelial growth factor (anti-VEGF) is used at one-month intervals (loading phase) in patients with DME^(2)^. This treatment regimen has shown good clinical outcomes for patients^(3)^.

VEGF is involved in the regulation of angiogenesis and neuroprotection in response to retinal ischemia and cell proliferation^(4)^. It is a potent, diffusible, endothelial-specific mitogen released in response to hypoxia; upon binding to the VEGF receptor-2, expressed by the vascular endothelium, it elicits angiogenesis and vascular hyperpermeability^(5)^. VEGF-A is also an important neurotrophic factor for the development of many cell types in the central nervous system, including glial and neural cells^(6)^. VEGF plays important roles in both the development and the maintenance of the vasculature. Activation of the VEGF/VEGFR-2 signaling pathway leads to the production of nitric oxide and prostacyclin I_2_, which are important in the regulation of the vascular tone and the coagulation cascade^(7)^. VEGF is also a primary angiogenic mediator in the proliferative phase of wound healing^(8)^.

The long-term consequences of intraocular VEGF sup pression on the retina have been a subject of interest and debate within the scientific and clinical communities. Interpretation of data from some animal and *in vitro* studies suggests that VEGF may be a survival factor for retinal neurons^(9)^. Therefore, on the one hand, the administration of anti-VEGF drugs inhibits angiogenesis, but on the other, it may also inhibit neuroprotective functions, causing toxic effects on retinal nerve structures.

The effect of intravitreal agents on the inner retinal layers is unclear. Some evidence suggests negative long-term effects of anti-VEGF therapy on the ganglion cell complex (GCC) with a significant decrease in the ganglion cell layer (GCL) compared with that in untreated eyes after long-term anti-VEGF therapy in patients with age-related macular degeneration (AMD)^(10)^. In contrast, Lalwani et al. reported that long-term intraocular VEGF inhibition has no neurotoxic effects^(11)^.

Spectral domain-optical coherence tomography (SD-OCT) has become a frequently used device for eva luating retinal layers, providing both qualitative and quan titative assessments. SD-OCT is a safe, noninvasive, standard diagnostic tool for assessing the presence of intraand subretinal fluid. In addition, SD-OCT is well suited for detecting subtle macular changes and early DME, as well as for monitoring changes after treatment. It provides cross-sectional, near-histological-resolution images of the entire macula and allows detailed analyses of each individual retinal layer^(12)^. SD-OCT exhibits high repeatability and reproducibility to measure macular and peripapillary retinal nerve fiber layer (RNFL) thicknesses. Quantitative measurements of the macular GCL and inner plexiform layer (IPL) are also possible^(13)^.

Our aim with this study was to investigate GCC changes in patients with DME treated with intravitreal injections of anti-VEGF (loading phase therapy with ranibizumab over three months) using SD-OCT to compare the measurements with those in the fellow eyes and with others in a control group of patients who were not treated with anti-VEGF. The secondary outcomes included modifications of best-corrected visual acuity (BCVA) and central macular thickness (CMT).

## METHODS

The local ethics committee of the Adana Training and Research Hospital approved the study, and all the procedures adhered to the tenets of the Declaration of Helsinki (270-2018). We obtained written informed consents from all participants.

This is a retrospective study and information on patients treated and followed up in the retina clinic for DME was collected from a database. The patients included in the study group were treatmentnaive and had no need for re-injections because the macular edema had regressed after three loading doses. The control group consisted of patients with DME who were untreated.

The inclusion criteria for both groups included age over 50 years, presence of DME that had been untreated, sufficiently clear ocular media, and adequate pupillary dilatation. We also included patients with metabolic-controlled diabetes and glycosylated hemoglobin (HbA1c) between 5.5% and 6.8%.

We excluded patients with a history of any disease other than diabetes, and those with any previous intervention for DME (intravitreal injection, laser photocoagulation, or vitrectomy), presence of diffuse macular edema, cystoid degeneration, epiretinal membrane, vi treomacular traction or adhesion, ocular surgery, glaucoma, or presence of media opacities that could distort image quality.

During the initial visit, the patients underwent a complete ocular examination including measurements of BCVA using a logMAR unit and slit-lamp biomicroscopy for anterior and posterior segments. We measured intraocular pressures (IOP) using a Goldmann applanation tonometer. The diagnosis of DME was based on the results of OCT and fluorescein angiography.

In the study group, all patients received 0.5 mg ranibizumab (Lucentis; Genentech USA, San Francisco, CA/ Novartis Ophthalmics, Basel, Switzerland) in a volume of 0.05 mL for each intravitreal injection. The interval between two consecutive doses injected into the same eye was at least four weeks. All patients completed a loading phase of three monthly intravitreal ranibizumab injections.

Prior to injections, the same surgeon applied the topical anesthetic Alcaine 0.5% (proparacaine HCL). The areas around the eye were sterilized with 10% povidone-iodine, and we applied 5% povidone-iodine in the conjunctival sac. The surgeon injected the intravitreal ranibizumab (0.5 mg or 0.05 ml) with a 30-gage needle through the inferotemporal pars plana at 3.5 mm posterior to the limbus. He inserted needle approximately 1.0 cm into the globe before injecting the solution. After the injection, he placed a sterile cotton swab on the injection site to prevent reflux of the medicine or vitreous. He administered antibiotic drops and examined the perfusion of the central retinal artery in each eye. The same surgeon performed all intravitreal injections.

Patients were evaluated monthly with measurements of BCVA, tonometry, and SD-OCT. We followed them up for 6 months after the loading phase. SD-OCT assessments of GCC and CMT were recorded at baseline, monthly for three months, and at six and nine months. We compared all OCT parameters to those at baseline in each eye and those eyes in the control group.

We used an SD-OCT apparatus (Retina Scan RS 3000 Advance, Nidek, CA, USA) to measure the thicknesses of the macula and GCC. We captured the OCT images based on the 12-mm horizontal macula line scans (1024 high-definition A-scans). Each image consisted of 120 averaged B-scans, with a 4-µm resolution. After capturing the macula line scan, the device automatically calculated the foveal thickness.

We defined the GCC thickness as the distance from the internal limiting membrane to the outer boundary of the IPL. OCTs were processed automatically to provide a thickness map of the GCC. We obtained the mean GCC thickness, and the mean superior hemiretinal and mean inferior hemiretinal GCC thicknesses. The same ophthalmologist performed all the measurements. We only included images with signal strength indexes>50.

### Statistical analysis

We performed a statistical analysis using the SPSS 20.0 statistics software package (IBM, Chicago, IL, USA). We processed continuous variables using the Kolmogorov-Smirnov test. All measurements were distributed normally except for the pre-injection visual acuity. We used the paired-sample Student t-test and repeated measures two-way analysis of variance test to compare the measurements between variables. We chose the age variable as a covariate to perform adjusted Bonferroni post hoc tests. We calculated Pearson correlation coefficients to determine the association between the variation in VA and GCC measurements. We considered all p-values<0.05 as statistically significant.

## RESULTS

We included 35 eyes of 35 patients (18 men, 17 women) in the study group. The mean patient age was 60 ± 4.38 years, and the mean HbA1c was 6.1 ± 0.6% in the study group. In the control group, we had 32 patients (17 men, 15 women). The mean age of the controls was 58 ± 3.2 years, and the mean HbA1c was 6.3 ± 1.1%. We found no statistically significant differences between the groups in terms of age, gender, and HbA1c levels.

In treated eyes, the mean BCVA before treatment was 0.70 ± 0.17, and it became 0.22 ± 0.20 after the loading dose (p<0.001) (Table 1). The mean IOP at baseline was 14 ± 1.2 mmHg. The mean IOPs were 15 ± 0.8 mmHg after the first month, 14.5 ± 1.4 after the second month, 13.4 ± 1.5 after the third month, and 12.7 ± 1.6 after the sixth month. We did not find significant IOP increases after each injection (p>0.05). In the study group, the CMT measured using OCT decreased significantly from baseline to the third month post-injection (p<0.05) (Table 1).

**Table 1:** Measurements at baseline, after injections, and at follow-up times.

		Baseline		1^st^ month		2^nd^ month		3^rd^ month	6^th^ month	9^th^ month		p^1^	p^2^	p^3^
Visual acuity (LogMAR)		0.70 ± 0.17		0.52 ± 0.15		0.40 ± 0.42		0.22 ± 0.20	0.30 ± 0.15	0.20 ± 0.12		<0.001	<0.001	<0.001
Macular thickness (µm)		440.57 ± 96.22		352.46 ± 81.70		323.21 ± 67.27		306.53 ± 61.58	290.3 5± 50.23	285.45 ± 42.05		<0.05	<0.001	<0.001
Mean GCC thickness (µm)		115.08 ± 16.72		112.01 ± 19.59		108.05 ± 14.78		101.05 ± 12.67	110.04 ± 15.07	113.12 ± 11.15		<0.05	>0.05	>0.05
Mean superior GCC (µm)		115.75 ± 26.24		110.85 ± 19.11		107.25 ± 15.23		103.96 ± 12.03	111.12 ± 12.04	114.21 ± 12.32		<0.05	>0.05	>0.05
Mean inferior GCC (µm)		110.42 ± 24.64		108.35 ± 20.07		104.85 ± 14.33		99.14 ± 13.31	108.33 ± 20.05	112.20 ± 11.15		<0.05	>0.05	>0.05

GCC= Ganglion cell complex; p difference between the groups; p^1^= difference between baseline and 3^rd^ month; p^2^= difference between baseline and 6^th^ month; p^3^= difference between baseline and 9^th^ month.

In treated eyes, the GCC (which contains the GCL and the IPL) differed significantly between baseline and the different follow-up values. The mean GCC thickness was 115.08 ± 16.72 µm before the first injection, and it became 101.05 ± 12.67 µm after the third injection (p<0.05) (Table 1). We found no significant effects of age and gender on macular thickness and GCC thickness. We found no correlations between the change in GCC thickness and the difference in visual acuity (r=0.045; p=0.742), or between the change in GCC thickness and the difference in macular edema (r=0.042; p=0.650).

In the untreated fellow eyes, the mean GCC and macular thicknesses in the follow-up were similar. We found no difference between the treated and untreated eyes in terms of the mean GCC thickness, but the macular thicknesses did differ between the groups (Table 2).

**Table 2:** Macular thickness comparison between the groups

Macular Thickness	Baseline		1^st^ month		2^nd^ month		3^rd^ month		6^th^ month		9^th^ month	p^1^	p^2^	p^3^
Treated eyes	440.57±96.22ᵃᵇ		352.46 ± 81.70ᵃ		323.21 ± 67.27ᵃ		306.53 ± 61.58ᵃ		290.35 ± 50.23ᵃᵇ		285.45 ± 42.05	<0.05	<0.001	<0.001
Untreated fellow eyes	235.46±56.10ᵃ		240.10 ± 45.42ᵃ		225.50 ± 38.65ᵃ		221.20 ± 45.56ᵃ		220.25 ± 36.38ᵃ		224.19 ± 25.43	>0.05	>0.05	>0.05
Control group	310.23±35.42ᵇ		325.25 ± 54.36		320.48 ± 45.23		318.62 ± 50.25		350.45 ± 35.30ᵇ			>0.05	<0.05	
p-value	<0.05^*^		<0.05^*^		<0.05^*^		<0.05^*^		<0.05^*^					

p difference between the groups; p^1^= difference between baseline and 3^rd^ month; p^2^= difference between baseline and 6^th^ month; p^3^= difference between baseline and 9^th^ month.

We found no different mean GCC thicknesses between the control and study groups until the sixth month follow-up measurement (p>0.05). However, the mean GCC thicknesses in the second and third months differed significantly between the treated patients and the controls, but this difference lost its significance after the sixth month with an increase in the mean GCC thickness in the treated patients. After the significant increase in mean macular thickness in the sixth month, the control group patients were initiated with anti-VEGF treatment, and we were not able to compare measurements for the ninth month follow-up in the study group (Table 3).

**Table 3:** Mean GCC thickness comparison between groups

GCC Thickness	Baseline	1^st^ month	2^nd^ month	3^rd^ month	6^th^ month	9^th^ month	p^1^	p^2^	p^3^
Treated eyes	115.08 ± 16.72	112.01 ± 19.59	108.05 ± 14.78ᵃ	105.05 ± 12.67ᵃ	110.04 ± 15.07	113.12 ± 11.15	<0.05	>0.05	>0.05
Untreated fellow eyes	114.10 ± 12.65	114.10 ± 15.32	113.56 ± 19.33	112.11 ± 14.35	111.25 ± 25.14	113.33 ± 25.65	>0.05	>0.05	>0.05
Control group	117.65 ± 11.15	117.64 ± 20.08	120.05 ± 18.45ᵃ	118.42 ± 21.22ᵃ	119.35 ± 15.13		>0.05	>0.05	
p-value	>0.05	>0.05	<0.05^*^	<0.05^*^	>0.05	>0.05			

GCC= Ganglion cell complex; p difference between the groups; p^1^= difference between baseline and 3^rd^ month; p^2^= difference between baseline and 6^th^ month; p^3^= difference between baseline and 9^th^ month.

## DISCUSSION

We aimed to evaluate the effect of intravitreal anti-VEGF injections on the macular GCC thickness, and we observed that the injections caused a decrease in macular GCC thickness.

Diabetic retinopathy (DR) affects both vascular and neural components^(14)^. There is a growing suspicion that DR may represent two separate pathologies affecting the retina^(14)^: one is a vasculopathy that affects the function and morphology of the major superficial vessels and capillary bed within the retina, leading to edema^(14)^. The other is a chronic neuropathy aggravated by inflammation, excitotoxicity, and oxidative stress, which lead to altered morphology of the retinal ganglion cells and neurotransmitter and synapse function changes that culminate in a loss of neurons^(14)^. Although vascular lesions and breakdown of the blood-retinal barrier are central to disease progression, neurodegeneration also makes an important contribution, and this process begins soon after the onset of diabetes^(14)^.

Thickening of the sensory retina can be detected using biomicroscopy but evaluating neuroglial changes in the retina is difficult. SD-OCT can produce high-resolution images showing the individual layers of the retina in great detail, and the analysis software can delineate and measure the thickness of the inner retinal layers^(13)^. In this study, we used SD-OCT to evaluate the macular GCC thickness, the CMT.

The GCC consists of the RNFL, the GCL, and the IPL. The ganglion cells are usually a monolayer in the peripheral retina, but within the macula, they are multilayered and are highly concentrated. Therefore, the macula may be considered the most valuable region for detecting early cell loss and changes^(15)^. Some studies have shown that the GCC thickness has the same diagnostic potential for glaucoma as the RNFL thickness^(16-17)^. However, GCC analysis is not limited to the differential diagnosis and management of glaucoma, but it can be applied to other neurological and retinal diseases^(13,18-20)^.

The GCC is affected by multiple diseases such as diabetes and macular degeneration, which cause retinal and choroidal structural changes^(10,21-22)^. Diabetes frequently causes retinopathies that lead to blindness by affecting the morphology of retinal ganglion cells. The axons in the retina with DR show morphologic changes such as irregular swelling and beading^(23)^. The nerve fiber layer comprises axons derived from ganglion cells. Defects in the RNFL have been reported clinically^(21)^, and basic research has shown ganglion cell apoptosis in DR^(24)^. Studies have shown tissue loss in the inner retina and thinning of the RNFL and retinal GCL in patients with both type 1 and type 2 diabetes, even before the onset of microvascular lesions^(19,22,25-28)^. Demir et al. found a nonsignificant loss of RNFL and GCC in patients with type 2 diabetes^(19)^. Van Dijk et al. reported a differential reduction in the thickness of the GCL and IPL combined and a smaller loss of the inner nuclear layer in patients with diabetes^(26)^.

VEGF-A is directly responsible for neoangiogenesis. The current DR treatments with anti-VEGF agents are directed at vascular pathologies and effectively reduce the vasogenic edema due to increased vascular permeability^(3,5)^. Retinal nerve structures changed in rats treated with six intravitreal injections of ranibizumab at weekly intervals, and repeated intravitreal injections of anti-VEGF-A monoclonal antibody led to the degeneration of their retinal ganglion cells^(9)^. Many studies have evaluated the effect of anti-VEGF agents on GCC and RNFL in patients with AMD^(10,20,29-30)^. Beck et al. found that the GCL was significantly thinner in the study eyes compared with the GCL in untreated fellow eyes^(10)^. In contrast, Zucchiatti et al. and Nishimura et al. reported a lack of adverse effects on any retinal layers, including in retinal ganglion cells, after intravitreal injections in eyes with AMD^(20,30)^.

In our study, we recruited patients with diabetes and treatment-naive DME. Injections were not continued after the loading doses. We found a significant decrease in GCC thickness after the third month. Without other injections, the GCC thicknesses increased until after the ninth month, when they almost reached their starting levels. Our study showed the GCC thicknesses decreased with anti-VEGF injections in patients with DME. The decreases in GCC thickness may be due to the toxic effects of anti-VEGF agents or may be the result of the natural course of the disease (diabetic neurodegeneration) independently of the drug. Although we found no differences between the treated and untreated eyes in terms of GCC thicknesses before treatment, the GCC thickness was lower in the treated eyes than in untreated eyes during the treatment, albeit without statistical significance. In addition, the GCC thicknesses were significantly lower in treated patients than in the control group during treatment. Based on our findings, we believe that the decrease in GCC thickness is due to the effect of the anti-VEGF agent, and in the least, the anti-VEGF agents may have accelerated the reduction in GCC thickness.

To the best of our knowledge, no similar studies have been published. Our study is the first to investigate the effect of anti-VEGF on GCC in patients with diabetes. Further randomized, controlled studies are necessary to ascertain whether our results were due to the natural history of the disease or secondary to the effect of anti-VEGF treatment.

Anti-VEGF agents are frequently used to treat vascular pathologies in cases of DR; however, little consideration has been given to treatment modalities to prevent neurodegeneration. In our study, we found a reduction in the GCC thickness after anti-VEGF treatment; the macular edema regressed, macular thickness decreased, and visual acuity increased. As a result, we think the decrease in GCC thickness was mostly caused by the effects of the anti-VEGF agents. Therefore, we cannot yet discard the anti-VEGF treatment for DME. However, novel therapies for DR should include neuroprotective agents. Further studies are needed to confirm our results and to develop neuroprotective treatment methods.

## References

[1] Cho YJ, Lee DH, Kim M. (2018). Optical coherence tomography findings predictive of response to
treatment in diabetic macular edema. J Int Med Res.

[2] James DG, Mitkute D, Porter G, Vayalambrone D. (2019). Visual Outcomes Following Intravitreal Ranibizumab for Diabetic
Macular Edema in a Pro Re Nata Protocol from Baseline: A Real-World
Experience. Asia Pac J Ophthalmol (Phila).

[3] Pham B, Thomas SM, Lillie E, Lee T, Hamid J, Richter T (2019). Anti-vascular endothelial growth factor treatment for retinal
conditions: a systematic review and meta-analysis. BMJ Open.

[4] Sondell M, Lundborg G, Kanje M. (1999). Vascular endothelial growth factor has neurotrophic activity and
stimulates axonal outgrowth, enhancing cell survival and Schwann cell
proliferation in the peripheral nervous system. J Neurosci.

[5] Miller JW, Le Couter J, Strauss EC, Ferrara N. (2013). Vascular endothelial growth factor a in intraocular vascular
disease. Ophthalmology.

[6] Storkebaum E, Lambrechts D, Carmeliet P. (2004). VEGF: once regarded as a specific angiogenic factor, now
implicated in neuroprotection. BioEssays.

[7] He H, Venema VJ, Gu X, Venema RC, Marrero MB, Caldwell RB. (1999). Vascular endothelial growth factor signals endothelial cell
production of nitric oxide and prostacyclin through flk-1/KDR activation of
c-Src. J Biol Chem.

[8] Nissen NN, Polverini PJ, Koch AE, Volin MV, Gamelli RL, DiPietro LA. (1998). Vascular endothelial growth factor mediates angiogenic activity
during the proliferative phase of wound healing. Am J Pathol.

[9] Nishijima K, Ng YS, Zhong L, Bradley J, Schubert W, Jo N (2007). Vascular endothelial growth factor-A is a survival factor for
retinal neurons and a critical neuroprotectant during the adaptive response
to ischemic injury. Am J Pathol.

[10] Beck M, Munk MR, Ebneter A, Wolf S, Zinkernagel MS. (2016). Retinal ganglion cell layer change in patients treated with
anti-vascular endothelial growth factor for neovascular age-related macular
degeneration. Am J Ophthalmol.

[11] Lalwani GA, Rosenfeld PJ, Fung AE, Dubovy SR, Michels S, Feuer W (2009). A variable-dosing regimen with intravitreal ranibizumab for
neovascular age-related macular degeneration: year 2 of the PrONTO
Study. Am J Ophthalmol.

[12] Badaró E, Novais E, Prodocimo LM, Sallum JM. (2014). Spectral-domain optical coherence tomography for macular
edema. Scientific World Journal.

[13] Lee WH, Jo YJ, Kim JY. (2018). Thickness of the Macula, Retinal Nerve Fiber Layer, and Ganglion
Cell-inner Plexiform Layer in the Macular Hole: The Repeatability Study of
Spectral-domain Optical Coherence Tomography. Korean J Ophthalmol.

[14] Barber AJ, Baccouche B. (2017). Neurodegeneration in diabetic retinopathy: potential for novel
therapies. Vision Res.

[15] Yang Z, Tatham AJ, Weinreb RN, Medeiros FA, Liu T, Zangwill LM. (2015). Diagnostic ability of macular ganglion cell inner plexiform layer
measurements in glaucoma using swept source and spectral domain optical
coherence tomography. PLoS One.

[16] Kim NR, Lee ES, Seong GJ, Kim JH, An HG, Kim CY. (2010). Structure-function relationship and diagnostic value of macular
ganglion cell complex measurement using Fourier-domain OCT in
glaucoma. Invest Ophthalmol Vis Sci.

[17] Celik E, Cakır B, Turkoglu EB, Doğan E, Alagoz G. (2016). Effect of cataract surgery on subfoveal choroidal and ganglion
cell complex thicknesses measured by enhanced depth imaging optical
coherence tomography. Clin Ophthalmol.

[18] Satue M, Obis J, Rodrigo MJ, Otin S, Fuertes MI, Vilades E (2016). Optical coherence tomography as a biomarker for diagnosis,
progression, and prognosis of neurodegenerative diseases. J Ophthalmol.

[19] Demir M, Oba E, Sensoz H, Ozdal E. (2014). Retinal nerve fiber layer and ganglion cell complex thickness in
patients with type 2 diabetes mellitus. Indian J Ophthalmol.

[20] Zucchıattı I, Cicinelli M, Parodi MB, Pierro L, Gagliardi M, Accardo A (2017). Effect of intravitreal ranibizumab on ganglion cell complex and
peripapillary retinal nerve fiber layer in neovascular age-related macular
degeneration using spectral domain optical coherence
tomography. Retina.

[21] Chihara E, Matsuoka T, Ogura Y, Matsumura M. (1993). Retinal nerve fiber layer defect as an early manifestation of
diabetic retinopathy. Ophthalmology.

[22] Carpineto P, Toto L, Aloia R, Ciciarelli V, Borrelli E, Vitacolonna E (2016). Neuroretinal alterations in the early stages of diabetic
retinopathy in patients with type 2 diabetes mellitus. Eye (Lond).

[23] Ishikawa H, Stein DM, Wollstein G, Beaton S, Fujimoto JG, Schuman JS. (2005). Macular segmentation with optical coherence
tomography. Invest Ophthalmol Vis Sci.

[24] Barber AJ, Lieth E, Khin SA, Antonetti DA, Buchanan AG, Gardner TW. (1998). Neural apoptosis in the retina during experimental and human
diabetes. Early onset and effect of insulin. J Clin Invest.

[25] Ng DS, Chiang PP, Tan G, Cheung CG, Cheng CY, Cheung CY (2016). Retinal ganglion cell neuronal damage in diabetes and diabetic
retinopathy. Clin Exp Ophthalmol.

[26] van Dijk HW, Kok PH, Garvin M, Sonka M, Devries JH, Michels RP (2009). Selective loss of inner retinal layer thickness in type 1
diabetic patients with minimal diabetic retinopathy. Invest Ophthalmol Vis Sci.

[27] Chhablani J, Sharma A, Goud A, Peguda HK, Rao HL, Begum VU (2015). Neurodegeneration in type 2 diabetes: evidence from
spectral-domain optical coherence tomography. Invest Ophthalmol Vis Sci.

[28] Frydkjaer-Olsen U, Soegaard Hansen R, Pedersen K, Peto T, Grauslund J. (2015). Retinal vascular fractals correlate with early neurodegeneration
in patients with type 2 diabetes mellitus. Invest Ophthalmol Vis Sci.

[29] Martinez-de-la-Casa JM, Ruiz-Calvo A, Saenz-Frances F, Reche-Frutos J, Calvo-Gonzalez C, Donate-Lopez J (2012). Retinal nerve fiber layer thickness changes in patients with
age-related macular degeneration treated with intravitreal
ranibizumab. Invest Ophthalmol Vis Sci.

[30] Nishimura T, Machida S, Harada T, Kurosaka D. (2012). Retinal ganglion cell function after repeated intravitreal
injections of ranibizumab in patients with age-related macular
degeneration. Clin Ophthalmol.

